# Supplementary material for: Elevated Fasting Blood Glucose Levels Are Associated with Worse Clinical Outcomes in COVID-19 Patients Than in Pneumonia Patients with Bacterial Infections
Source: Pathogens. 2022 Aug 10;11(8):902. doi: 10.3390/pathogens11080902 (PMC9416056; doi:10.3390/pathogens11080902)
Supplement: Supplementary file 1 [file pathogens-11-00902-s001.zip › pathogens-1735543-supplementary.pdf]

Supplemental Appendix

Elevated Fasting Blood Glucose Levels are Associated with Worse Clinical Outcomes in COVID-19 Patients than in Pneumonia Patients with Bacterial Infections

Wenjun Wang [1,2,3], Zhonglin Chai [4], Mark E Cooper [4], Paul Z Zimmet [4], Hua Guo [5], Junyu Ding [5], Feifei Yang [1,2,3], Xixiang Lin [1,2,3], Xu Chen [1,2,3], Xiao Wang [1,2,3], Qin Zhong [1,2,3], Zongren Li [1,2,3], Peifang Zhang [6], Zhenzhou Wu [6], Xizhou Guan [5,*], Lei Zhang [4,,7,8,9,*] and Kunlun He [1,2,3,*]

[1] Key Laboratory of Ministry of Industry and Information Technology of Biomedical Engineering and Translational Medicine, Chinese PLA, General Hospital, Beijing 100853, China

[2] Beijing Key Laboratory for Precision Medicine of Chronic Heart Failure, Chinese PLA, General Hospital, Beijing 100853, China

[3] Medical Big Data Research Center, Chinese PLA, General Hospital, Beijing 100853, China

[4] Central Clinical School, Faculty of Medicine, Monash University, Melbourne 3004, Australia

[5] Department of Pulmonary and Critical Care Medicine, Chinese PLA, General Hospital, Beijing 100853, China

[6] BioMind Technology, Zhongguancun Medical Engineering Center, 10 Anxiang Road, 8th Floor, Beijing 100872, China

[7] China-Australia Joint Research Center for Infectious Diseases, School of Public Health, Xi'an Jiaotong University Health Science Center, Xi'an 710061, China

[8] Artificial Intelligence and Modelling in Epidemiology Program, Melbourne Sexual Health Centre, Alfred Health, Melbourne 3004, Australia

[9] Department of Epidemiology and Biostatistics, College of Public Health, Zhengzhou University, Zhengzhou 450001, China

* Correspondence: kunlunhe@301hospital.com.cn (K.H.); lei.zhang1@monash.edu (L.Z.); guanxizhou@plagh.com (X.G.)

Definition of clinical symptoms and complications

1. Acute respiratory distress syndrome was defined as the acute onset of hypoxemia (PaO2/FiO2 ≤ 200) with bilateral infiltrates at x-rays and no evidence of left atrial hypertension [48].

2. Shock was defined as a clinical state of cellular and tissue gypoxia caused by reducing oxygen delivery and utilization or increasing oxygen consumption[49].

3. Acute heart failure was defined as a clinical syndrome characterized by typical symptoms (e.g. breathlessness, ankle swelling and fatigue) that may be accompanied by signs (e.g. elevated jugular venous pressure, pulmonary crackles and peripheral oedema) caused by a structural and/or functional cardiac abnormality [50].

4. Acute kidney injury was defined AKI was diagnosed by reference (exclusively) to SCr level, thus by an SCr increase ≥ 0.3 mg/dl (≥ 26.5 μmol/L) within 48 h, or an increase to ≥ 1.5-fold the baseline value, known or presumed to have developed within the prior 7 days [51].

5. Acute myocardial injury was defined as blood levels of cardiac biomarkers (hs-TNI) above the 99th-percentile upper reference limit, regardless of new abnormalities in electrocardiography and echocardiography[52].

6. Respiratory failure was defined as a failure to maintain adequate gas exchange and is characterized by abnormalities of arterial blood gas tensions [53].

7. Acute hepatitis was defined as a wide variety of clinical state characterized by elevated liver function indices caused by acute inflammation of hepatic parenchyma or injury to hepatocytes[54].

8. Acute liver failure was defined as severe acute liver injury with encephalopathy and impaired synthetic function (INR ≥ 1.5) in patients without pre-existing liver disease and with duration of < 26 weeks[55].

9. Cough, Fatigue, Diarrhea, Chest tightness and Shortness of breath symptoms of pneumonia were defined in accordance with the American Lung Association[56].

10. According to the Chinese guidelines for the prevention and control of type 2 diabetes (2017 edition)[26], we used the FBG level as the key parameter to stratify the groups in our analysis. We categorized patients into 3 groups by their FBG level at admission, including FBG <6.1 mmol/L, FBG 6.1-6.9 mmol/L, and FBG >=7.0 mmol/L. people are considered to be high FBG levels which is 6.1-6.9 mmol/L and then is potential risk of diabetes when FBG >=7.0 mmol/L.

Table S1. The participant criterias of inclusion, exclusion and clinical discharge. (The criterias were defined according to the guidline; COVID-19: Diagnosis and Treatment Protocol for Novel Coronavirus Pneumonia (Trial Version 7); Pneumonia patients with sole bacterial infection or concurrent bacterial and fungal infections: Diagnosis and Treatment Guideline for Community-Acquired Pneumonia (The Version 2016)).

Participants	Inclusion criteria	Exclusion criteria	Clinical discharge criteria
COVID-19	Any of the following:<br>1.a positive result for detection of SARS-CoV-2 nucleic acid fragment by the revers transcription polymerase chain reaction; 2.detection of seral IgM and IgG antibodies to SARS-Cov-2.	Any of the following:<br>1.patients transferred to another medical institution during hospitalization; 2.patients who were admitted to the hospital multiple times; 3.patients younger than 18 years old.	All of the following：<br>1.body temperature returned to normal (<37.5^{o}C) for three consecutive days; 2.respiratory symptoms improved substantially; 3.pulmonary imaging showed an obvious reduction in inflammation; and 4.two consecutive laboratory tests showing negative detection of SARS-Cov-2 by RT-PCR, each at least 24 hours apart.
Pneumonia patients with sole bacterial infection or concurrent bacterial and fungal infections	Any of the following:<br>1.clinical manifestation: clinical symptom of fever; or clinical symptom of cough, expectoration with or without purulent sputum, chest pain, short of breath, or hemoptysis; or the count of peripheral white blood cells >10 or <4 (10^9/L); 2.clinical signs of pulmonary consolidation or moist rales.	Any of the following :<br>1.patients younger than 18 years old; 2. patients with pneumonia with infection by pathogen(s) other than bacterium or bacterium and fungus, such as virus, mycoplasma, chlamydia, parasite and abiotic etiology.	All of the following ：<br>1.body temperature returned to normal (<37.5^{o}C) for 24 hours; 2.clinical manifestation and complications have been improved.

Table S2 Basic demographic characteristics, signs and symptoms, comorbidities, laboratory findings, treatment and clinical outcomes of patients with pneumonia. (The participants with missing data for FBG levels on admission within 72 hours were not included in these calculations for patients)

**Variable**	**COVID-19 (FBG) (N=2366)**			**Pneumonia with bacterial infection (FBG) (N=1630)**			**Pneumoia with bacterial and fungal infection (FBG) (N=1988)**		
	<6.1mmol/L (N=1904)	6.1-6.9 mmol/L (N=152)	>=7.0 mmol/L (N=310)	<6.1 mmol/L (N=1015)	6.1-6.9 mmol/L (N=188)	>=7.0 mmol/L (N=427)	<6.1 mmol/L (N=985)	6.1-6.9 mmol/L (N=302)	>=7.0 mmol/L (N=701)
**Demographic characteristics at admission**									
Age(yr) —median (IQR)	59.0(48.0,67.0)	65.0(55.8,71.0)	64.5(57.0,71.0)	57.0(45.5,67.0)	62.0(49.8,75.0)	62.0(50.5,74.0)	64.0(50.0,78.0)	67.0(54.0,81.0)	66.0(53.0,80.0)
<45 —no. (%)	370(19.4)	12(7.9)	9(2.9)	234(23.1)	37(19.7)	66(15.5)	167(16.9)	35(11.6)	96(13.7)
45-59 —no. (%)	604(31.7)	41(27.0)	94(30.3)	329(32.4)	47(25.0)	124(29.0)	227(23.1)	67(22.2)	154(22.0)
60-74 —no. (%)	741(38.9)	70(46.0)	154(49.7)	321(31.6)	54(28.7)	132(30.9)	290(29.4)	79(26.2)	197(28.1)
>74 —no. (%)	189(9.9)	29(19.1)	53(17.1)	131(12.9)	50(26.6)	105(24.6)	301(30.6)	121(40.1)	254(36.2)
Male gender —no. (%)	921(48.4)	88(57.9)	162(52.3)	690(68.0)	136(72.7)	302(70.7)	636(64.6)	212(70.2)	512(73.2)

Respiratory rate>20 min									
—no. (%)	515(27.1)	54(35.8)	123(39.7)	64(6.8)	20(11.8)	62(15.3)	141(15.4)	48(17.1)	136(20.9)
Pulse rate >100 per min									
—no. (%)	293(15.4)	24(15.9)	64(20.6)	90(9.6)	23(13.6)	84(20.7)	124(13.6)	46(16.4)	184(28.2)
Systolic blood pressure>=140 mmHg									
—no. (%)	434(24.9)	46(32.9)	112(39.0)	239(25.6)	51(30.2)	154(38.0)	216(23.7)	91(32.6)	197(30.2)
Diastolic blood pressure>=90mmHg									
—no. (%)	421(24.1)	32(22.9)	60(20.9)	145(15.5)	23(13.6)	66(16.3)	104(11.4)	33(11.8)	81(12.4)
**Signs and Symptoms —no. (%)**									
Fever	39(2.1)	14(9.3)	25(8.1)	52(6.6)	25(18.0)	56(16.3)	73(9.5)	41(17.0)	107(19.4)
Axillary temperature	36.5(36.3,36.7)	36.5(36.2,36.8)	36.5(36.3,36.8)	36.5(36.3,36.8)	36.6(36.5,37.1)	36.6(36.4,37.1)	36.6(36.4,36.9)	36.6(36.4,37.2)	36.7(36.5,37.3)
Cough	75(3.9)	3(2.0)	14(4.5)	280(27.6)	56(29.8)	99(23.2)	378(38.4)	111(36.8)	206(29.4)
Fatigue	713(37.5)	61(40.1)	136(43.9)	25(2.5)	9(4.8)	15(3.5)	36(3.6)	11(3.6)	22(3.1)

Diarrhea	51(2.7)	7(4.6)	6(1.9)	6(0.6)	1(0.5)	4(0.9)	10(1.0)	4(1.3)	10(1.4)
Chest tightness	222(11.7)	16(10.5)	51(16.4)	64(6.3)	23(12.2)	47(11.0)	84(8.5)	24(8.0)	65(9.3)
Shortness of breath	452(23.7)	34(22.4)	89(28.7)	76(7.5)	20(10.6)	33(7.7)	94(9.5)	28(9.3)	64(9.1)
**Pre-existing comorbidities —no. (%)**									
Coronary heart disease	98(5.2)	15(9.9)	37(11.9)	119(11.7)	42(22.3)	123(28.8)	199(20.2)	84(27.8)	198(28.2)
Cancer	23(1.2)	3(2.0)	12(3.9)	379(37.3)	54(28.7)	99(23.2)	288(29.2)	85(28.1)	145(20.7)
Chronic bronchitis	44(2.3)	2(1.3)	5(1.6)	133(13.1)	23(12.2)	32(7.5)	175(17.8)	54(17.9)	70(10.0)
Cerebrovascular disease	55(2.9)	15(9.9)	24(7.7)	100(9.8)	26(13.8)	78(18.3)	202(20.5)	67(22.2)	193(27.5)
Chronic kidney disease	39(2.0)	4(2.6)	12(3.9)	201(19.8)	51(27.1)	127(29.7)	243(24.7)	74(24.5)	224(31.9)
Chronic obstructive pulmonary disease	18(0.9)	2(1.3)	3(1.0)	36(3.5)	16(8.5)	24(5.6)	89(9.0)	21(7.0)	56(8.0)
Diabetes	101(5.3)	47(30.9)	181(58.4)	97(9.6)	34(18.1)	193(45.2)	115(11.7)	61(20.2)	283(40.4)
Hepatitis	25(1.3)	2(1.3)	4(1.3)	37(3.6)	3(1.6)	7(1.6)	31(3.1)	11(3.6)	18(2.6)
Hypertension	492(25.8)	65(42.8)	152(49.0)	288(28.4)	68(36.2)	202(47.3)	344(34.9)	132(43.7)	340(48.5)

Laboratory findings —median (IQR)

C-reactive protein (mg/L)	1.8(0.7,5.4)	5.0(1.6,32.1)	5.3(1.6,36.1)	10.1(3.2,44.7)	24.3(4.9,78.1)	42.0(8.4,99.9)	22.4(4.9,68.5)	37.8(11.4,95.9)	46.0(15.4,118.3)
D-dimer (mg/L)	0.3(0.2,0.7)	0.6(0.3,1.0)	0.6(0.3,1.3)	0.7(0.4,1.7)	1.1(0.5,2.5)	1.3(0.6,3.3)	1.3(0.6,2.5)	1.9(0.9,3.3)	2.0(1.0,4.0)
White blood cell count (109/L)	5.6(4.6,6.8)	6.2(4.8,7.8)	6.3(5.1,8.2)	6.5(5.1,8.6)	8.4(6.0,11.3)	8.6(6.3,11.8)	7.3(5.2,10.2)	8.1(6.0,10.9)	9.5(6.9,13.8)
Lymphocyte	0.3(0.2,0.3)	0.2(0.2,0.3)	0.2(0.1,0.3)	0.2(0.1,0.3)	0.1(0.1,0.2)	0.1(0.1,0.2)	0.2(0.1,0.3)	0.1(0.1,0.2)	0.1(0.1,0.1)
Neutrophils	0.6(0.5,0.7)	0.6(0.6,0.7)	0.7(0.6,0.8)	0.7(0.6,0.8)	0.8(0.7,0.9)	0.8(0.7,0.9)	0.7(0.6,0.8)	0.8(0.7,0.8)	0.8(0.8,0.9)
Lactate dehydrogenase (IU/L)	171.9(148.9,205.0)	194.4(163.3,242.7)	199.0(167.3,282.9)	173.4(143.8,222.0)	194.3(155.8,254.8)	202.4(159.5,282.1)	194.7(153.7,261.1)	212.1(164.3,278.6)	232.0(174.3,357.3)
Thrombinogen time (s)	12.8(12.2,13.5)	12.8(12.2,13.5)	12.7(12.2,13.7)	15.9(15.2,16.8)	15.5(14.8,16.3)	15.5(14.6,16.4)	15.7(14.9,16.7)	15.7(14.9,16.7)	15.6(14.7,16.7)
Monocyte	0.1(0.1,0.1)	0.1(0.1,0.1)	0.1(0.1,0.1)	0.1(0.1,0.1)	0.1(0.1,0.1)	0.1(0.0,0.1)	0.1(0.1,0.1)	0.1(0.0,0.1)	0.1(0.0,0.1)
Total bilirubin (μmol/L)	9.6(7.3,12.3)	9.5(7.4,12.0)	9.5(7.2,12.8)	9.8(7.1,14.0)	9.9(7.0,14.3)	10.3(7.2,15.6)	9.2(6.9,13.7)	11.3(7.9,16.9)	10.7(7.3,17.1)
Direct bilirubin (μmol/L)	3.3(2.5,4.3)	3.5(2.7,4.7)	3.6(2.6,5.0)	3.0(2.1,4.7)	3.0(1.9,5.2)	3.2(2.1,5.7)	3.1(2.2,5.0)	4.1(2.8,6.6)	4.0(2.5,6.5)

Albumin (g/L)	38.4(35.6,40.7)	37.0(33.2,40.1)	36.9(33.7,39.7)	53.7(48.0,58.3)	53.9(47.6,58.5)	52.8(46.8,57.0)	50.5(46.1,56.0)	50.7(47.0,56.4)	51.5(45.7,56.0)
Fibrinogen (g/L)	2.9(2.6,3.3)	3.2(2.9,3.8)	3.2(2.8,3.6)	4.1(3.2,5.4)	4.6(3.7,6.2)	5.0(3.8,6.5)	4.4(3.5,5.6)	4.5(3.5,5.9)	5.0(3.7,6.2)
Creatinine (μmol/L)	64.1(55.1,75.3)	67.1(55.7,76.2)	62.7(53.6,75.0)	72.1(60.0,88.1)	76.6(61.7,111.2)	75.1(60.0,111.1)	70.5(55.6,92.0)	69.8(54.1,95.7)	74.8(55.3,108.6)
Creatine kinase (U/L)	51.6(37.4,72.5)	50.0(33.2,70.8)	45.6(33.7,73.7)	59.8(37.0,98.3)	64.8(35.9,123.0)	68.9(41.6,161.0)	47.5(28.4,88.1)	53.4(27.6,113.9)	63.4(31.7,140.0)
Creatine kinase-MB (IU/L)	8.4(6.9,10.6)	8.8(7.5,11.8)	9.7(7.8,12.8)	2.6(1.0,13.6)	1.7(0.8,3.8)	1.8(0.9,4.1)	1.9(1.0,7.2)	1.5(1.0,3.2)	1.8(1.0,3.8)
Blood glucose	4.7(4.4,5.1)	6.5(6.2,6.7)	8.8(7.7,11.1)	4.9(4.4,5.4)	6.5(6.3,6.7)	9.1(7.8,11.3)	5.0(4.5,5.5)	6.5(6.3,6.7)	9.2(7.8,11.6)
Cystatin C (mg/L)	0.9(0.8,1.1)	1.0(0.9,1.1)	1.0(0.8,1.1)	1.1(0.9,1.4)	1.2(0.9,3.3)	1.4(1.0,3.0)	1.2(1.0,1.6)	1.1(0.9,1.8)	1.3(1.0,1.9)
Platelets count(109/L)	219.0(182.0,267.5)	221.0(179.2,276.5)	223.0(176.5,280.5)	218.5(168.0,276.2)	205.5(153.0,280.0)	196.0(149.0,262.0)	205.0(146.0,280.0)	197.5(139.2,261.8)	192.0(133.0,257.2)
**Complications during hospitalization —no. (%)**									
Acute respiratory distress syndrome	6(0.3)	1(0.7)	13(4.2)	0(0.0)	0(0.0)	3(0.7)	1(0.1)	1(0.3)	3(0.4)
Acute myocardial injury/failure	24(1.3)	3(2.0)	10(3.2)	235(23.1)	72(38.3)	175(41.0)	368(37.4)	129(42.7)	324(46.2)

Acute hepatitis/liver failure	27(1.4)	4(2.6)	11(3.5)	218(21.5)	45(23.9)	115(26.9)	300(30.5)	110(36.4)	232(33.1)
Respiratory failure	17(0.9)	4(2.6)	19(6.1)	28(2.8)	14(7.5)	44(10.3)	115(11.7)	52(17.2)	159(22.7)
Shock	7(0.4)	2(1.3)	5(1.6)	6(0.6)	1(0.5)	4(0.9)	18(1.8)	9(3.0)	33(4.7)
Acute kidney injury	4(0.2)	1(0.7)	2(0.7)	207(20.4)	48(25.5)	132(30.9)	271(27.5)	89(29.5)	248(35.4)
**outcomes**									
The length of stady (days) —median (IQR)	12.0(8.0,18.0)	15.0(10.0,21.0)	15.0(10.0,22.0)	14.0(10.0,20.0)	13.0(8.0,19.0)	14.0(9.0,20.0)	19.0(13.0,28.0)	21.0(13.0,29.0)	20.0(13.0,31.0)
ICU —no. (%)	43(2.3)	11(7.2)	38(12.3)	40(3.9)	13(6.9)	35(8.2)	57(5.8)	24(8.0)	73(10.4)

Table S3 (Supplementary analysis of Figure2) Changes in Fasting Blood Glucose in three pneumonia patient groups at admission, during hospitalization and at discharge (The results below were presented as P-value by Nemenyi test).

**FBG levels (mmol/L) during hospitalization / at admission**	**< 6.1**	**6.1-6.9**	**>=7.0**	**FBG levels (mmol/L) at discharge / at admission**	**< 6.1**	**6.1-6.9**	**>=7.0**
COVID-19	<0.001**	<0.001**	0.588	COVID-19	<0.001**	<0.001**	0.031*
Pneumonia of bacterial infection	<0.001**	<0.001**	0.029*	Pneumonia of bacterial infection	0.755	<0.001**	<0.001**
Pneumonia of bacterial and fungal infection	<0.001**	<0.001**	<0.001**	Pneumonia of bacterial and fungal infection	0.002*	<0.001**	0.003*

*Compared with the FBG levels at admission, the P-value is between 0.05 and 0.001; **Compared with the FBG levels at admission, the P-value < 0.001;

Table S4 (Supplementary analysis of Figure3) Adjusted interaction of FBG levels and pneumonia on complications and hospital outcomes in patients (The results below were presented as P-value by interaction test).

	Bacterial infection		Concurrent infections	
	FBG 6.1-6.9 mmol/L	FBG >=7.0 mmol/L	FBG 6.1-6.9 mmol/L	FBG >= 7.0 mmol/L
Acute Respiratory distress syndrome	0.999	0.987	0.767	0.291
Acute myocardial injury / heart failure	0.452	0.899	0.908	0.622
Acute hepatitis / liver failure	0.468	0.152	0.511	0.029*
Respiratory failure	0.774	0.099	0.305	0.0017*
Shock	0.273	0.171	0.376	0.322

Acute kidney injury	0.309	0.749	0.463	0.627
The length of stay	0.0927	0.0212*	0.575	0.334
Intensive care unit	0.266	0.0014*	0.058	<0.001**

*Compared with COVID-19 and FBG<6.1 mmol/L, the P-value is between 0.05 and 0.001; **Compared with COVID-19 and FBG<6.1 mmol/L, the P-value < 0.001; Parameters were adjusted, including age, sex and pre-existing comorbidities (chronic obstructive pulmonary disease, chronic kidney disease, cerebrovascular disease, coronary heart disease, cancer, chronic bronchitis, hepatitis and hypertension
